# Efficient and participatory design of scale-appropriate agricultural machinery workshops in developing countries: A case study in Bangladesh

**DOI:** 10.1016/j.deveng.2019.100046

**Published:** 2020

**Authors:** Ellerbe Somers Gregg, Jonathan Colton, Md Abdul Matin, Timothy J. Krupnik

**Affiliations:** a Georgia Institute of Technology, Schools of Mechanical Engineering, Industrial Design, International Affairs, Atlanta, GA, 30332-0405, USA; b International Maize and Wheat Improvement Center, Sustainable Intensification Program, H 10/B, R53. Gulshan 2, Dhaka, 1212, Bangladesh; c Farm Machinery and Postharvest Process Engineering Division, Bangladesh Agricultural Research Institute, Joydebpur, Gazipur, 1701, Bangladesh

**Keywords:** Participatory action research, Agricultural machinery, Two-wheeled tractor, Factory design, Fabrication

## Abstract

Smallholder farmers provide the foundation for food security in South Asia. However, increasing seasonal labor scarcity caused by rural out-migration has resulted in growing agricultural labor costs, presenting challenges to cash-constrained smallholder farmers that hire manual labor for land preparation, sowing, harvest and post-harvest operations. Technological innovations in small-scale agricultural machinery appropriate for the small field sizes and limited resource endowments of South Asia's farmers have been proposed as a potential solution to this problem. An increasing number of development initiatives also promote rural entrepreneurial approaches to mechanization, whereby smallholder farmers can access and use machinery in their own fields on an affordable fee-for-service basis offered by machinery owners. This approach reduces capital constraints for smallholder farmers while enabling entrepreneurs who can afford equipment to enter into business serving stallholder farmers as clients. This approach is now widely practiced in Bangladesh, where machinery entrepreneurs play a crucial role in providing access to productive technologies for smallholder farmers who could not otherwise afford direct purchase of labor- and cost-saving machinery. In order to maintain low machinery purchase costs for emerging yet capital constrained rural entrepreneurs, while also assuring high quality standards, cost-effective domestic production of agricultural machinery is increasingly championed as an important long-term national development objective. With no safety standards or guidelines for best production practices, the few manufacturing workshops that exist within Bangladesh operate inefficiently and without clear rationalization of manufacturing processes. Haphazard copying of prototypes or imported available machinery is common. This leads to inefficient production and poor product quality in an emerging but potentially highly beneficial industry. This paper addresses these problems and presents a case study to increase machinery manufacturers' capacity while improving manufacturing operations and workplace safety through equipment selection, workshop layout, and usability.

Janata Engineering (JE) is a small-scale machinery manufacturing enterprise in Bangladesh, specializing in two-wheel tractor attachments such as bed planters, local derivations of power-tiller operated seeders, and other equipment for planting, irrigating, and processing crops. JE was expanding and setting up a second factory for which the authors provided assistance on its design. Our research question was whether participatory action research (PAR) supported by empirical data could provide improved factory design in terms of functionality, safety and human interactions, when compared with conventional approaches driven by technical efficiency concerns alone. Using PAR, we developed a number of alternative process and layout recommendations for JE to increase the efficiency of labor and machinery through improved workflow, throughput, and output. While immediately useful for JE, the process and protocols proposed in this paper are relevant for emerging agricultural machinery manufacturers in Bangladesh and more widely in South Asia.

## Highlights

• A method is presented for factory design for small agricultural machinery manufacturers in developing countries.
• Participatory action research improves design relevancy for emerging-world agricultural machinery manufacturers.
• Lowering overall handling distance reduces labor, probability of product damage and costs, by increasing throughput.

## 1 Introduction

The implications of agricultural mechanization have been a subject of scholarly debate for several decades (Mellor, 1973; Agarwal, 1981; Sison et al., 1985; Biggs et al., 2011; Kienzle et al., 2013). Although concerns regarding rural labor displacement were widely voiced in the early literature (Mellor, 1973; Smith and Gascon, 1979; Agarwal, 1981; Sison et al., 1985), rural communities in sub-Saharan Africa and South Asia are increasingly witnessing out-migration as farmers leave their homes in search of more remunerative employment (de Haan, 2002; Gartaula et al., 2012; Mendola, 2012; Zhang et al., 2014). This has created growing agricultural labor scarcity challenges and has refocused attention on agricultural mechanization in development research and policy (FAO, 2008; Collette et al., 2011; Mrema et al., 2014). Farm mechanization can assist in increasing the speed and precision of crop establishment, intercultural operations, harvest and post-harvest activities, while also reducing drudgery (Biggs et al., 2011; Kienzle et al., 2013; Baudron et al., 2015). In the context of increasingly labor-scarce agricultural systems in developing nations, appropriate use of mechanization can also increase smallholder farmers’ total production efficiency and returns to costs, while also alleviating labor constraints (Biggs et al., 2011; Mottaleb et al., 2016).

Contemporary development initiatives focusing on farm mechanization therefore increasingly point to the need for ‘scale-appropriate’ mechanization that makes use of affordable equipment custom-designed for the small fields and low-resource endowments of smallholder farming systems (Krupnik et al., 2013). These initiatives also tend to popularize rural entrepreneurial approaches to expanding smallholders' access to mechanization (USAID, 2016). Use of ‘service provision’ arrangements, whereby farmers can access and use machinery on their own fields on an affordable fee-for-service basis offered by machinery owners, is increasingly popular (Mottaleb et al., 2016). Machinery service provision can therefore overcome some of the capital constraints to machinery purchase for smallholder farmers, while enabling entrepreneurs who can afford equipment earn extra income after tending to their own fields through business endeavors serving smallholder farmers clients (Keil et al., 2016).

Agricultural machinery service provision is widely practiced in Bangladesh (Mandal, 2014), which despite having one of the world's densest rural populations at 1252 people km^−2^ (World Bank, 2018) is faced with rural agricultural communities that are increasingly labor constrained as farmers migrate to seek seasonal and full time alternative employment (Zhang et al., 2014). Rural out-migration is particularly popular among youth, who appear to be progressively less interested in engaging in farming as a livelihood pursuit (USAID, 2016). In this context, machinery entrepreneurs play a crucial role in providing access to labor- and cost-saving technologies for smallholder farmers who could not otherwise afford to purchase machinery (Mandal, 2014; Mottaleb et al., 2016). Yet when considered as a business, rural machinery service provision can also be prestigious, incentivizing youth who are able to purchase machinery to remain involved in agriculture and in rural communities (USAID, 2016).

Small-scale, two-wheel ‘hand tractors’ and associated 8–16 horsepower engines have been prevalent Bangladesh since the mid-1990s, and are accessible to smallholders through service provision. The most common use of agricultural machinery – most of which is imported from China – has been for powering irrigation pumps or primary land preparation using power tillers that can be attached to two-wheel tractors (Mottaleb et al., 2016). Work conducted by the Bangladesh Agricultural Research Institute (BARI), the International Maize and Wheat Improvement Center (CIMMYT), and Cornell University since the mid-2000s has conversely focused on developing scale-appropriate agricultural machinery that improves the precision and speed of crop establishment, in addition to land preparation, and that can be attached to two-wheel tractors (2WTs). Because of these efforts, a suite of 2WT attachable land-preparation and crop establishment implements have been engineered, including zero-tillage seed drills, power tiller operated seeders, and bed planters. Depending on the type of seed meter and drill, these machines can be used to establish rice, wheat, maize and leguminous crops, among others (Krupnik et al., 2013).

Domestic production of these implements is however nascent and is challenged by high production costs and poor manufacturing knowledge and standards. Bangladeshi-produced agricultural machinery also pales in comparison to the large volume of equipment imported from China, India, and South East Asia. While such imports help to make equipment more available, high shipping costs necessitate governmental subsidies to reduce prices and facilitate wide-spread purchase and adoption (Mandal, 2014; Mottaleb et al., 2016). If manufacturing costs can be kept low, domestic machinery production may conversely help to alleviate the high-costs of machinery import and need for governmental intervention (Krupnik et al., 2013; Mandal, 2014).

Yet with no guidelines for best production practices, limited practical experience, and no safety regulations, the few manufacturing workshops that exist within Bangladesh tend to operate inefficiently and without clear planning and rationalization of manufacturing processes. Because many manufacturers lack basic numeracy and engineering education, haphazard copying of imported machinery and prototypes is common. The result is inefficient production and poor product quality in an emerging but potentially highly beneficial industry (Krupnik et al., 2013). Many manufacturers also lack proper safety standards, a result of a lack of production protocols or guidelines for best practices and minimum safety standards (United States Congress, 2014).

This paper presents a case study (Gregg, 2015) of a machinery manufacturer in Bangladesh producing 2WT attachable bed planters as described in Krupnik et al. (2013). Our aims were to identify ways to increase machinery manufacturers’ capacity while improving manufacturing operations and workplace safety through equipment selection, workshop layout, and usability. As a locally-owned, small-scale agricultural machinery manufacturer in Bangladesh, Janata Engineering (JE) is representative of many small-scale and emerging machinery manufacturing enterprises in South Asia. JE focuses on manufacturing innovative land preparation and crop establishment implements attachable to 2WTs, including bed planters, derivations of power-tiller operated seeders, as well as other equipment for planting, irrigating, and harvesting cereal crops. JE was expanding and setting up a second factory for which the authors provided assistance on its design. Our research question was whether a participatory action research (PAR) approach supported by empirical data would provide better factory designs in terms of functionality, safety and human interactions, in comparison to conventional and technically-driven engineering approaches alone, i.e., designs produced by factory or product developers without input from technical experts or employees. Using PAR, we developed a number of alternative process and layout recommendations for JE to increase the efficiency of labor and equipment through improved workflow, throughput, and output. While immediately useful for JE, the process and protocols proposed in this paper are relevant for emerging agricultural machinery manufacturers in Bangladesh and more widely in South Asia.

## 2 Two-wheel tractor attachable bed planters

**Fig. 1:**
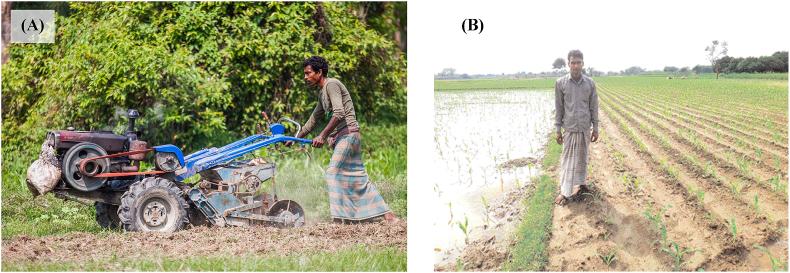
(A) Two-wheeled tractor with an attachable bed planter in Bangladesh (courtesy Ranak Martin). (B) Bed planted maize (right) compared to flat planted and waterlogged maize (left) following a storm event Bangladesh (photo from Krupnik et al., 2013).

**Fig. 2:**
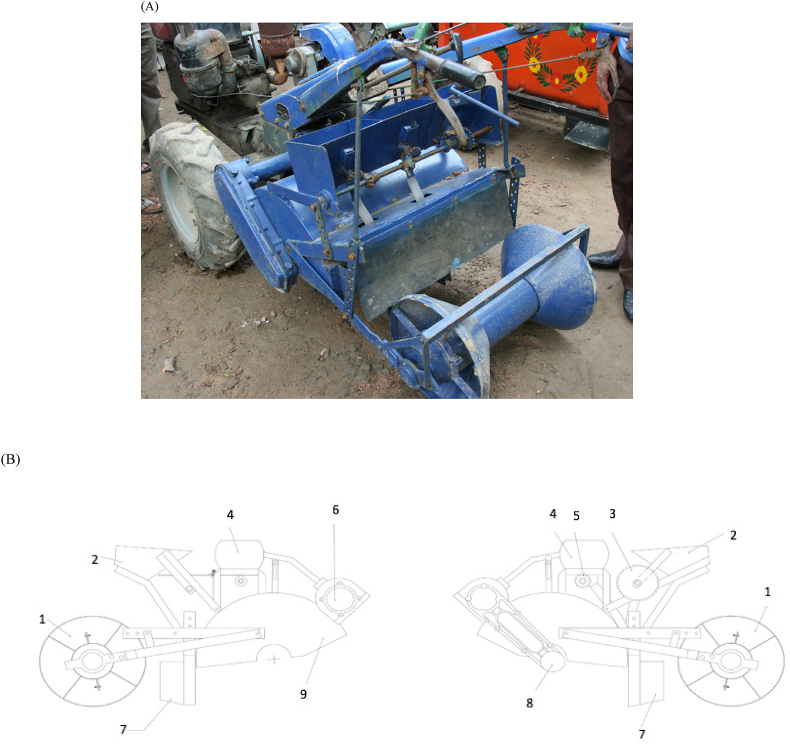
(A) Rear view of a two-wheel tractor bed planter displaying the bed shape former. (B) Assembly drawing: 1. Bed shape former; 2. Seed box; 3. Seed metering system; 4. Fertilizer box; 5. Fertilizer metering system; 6. Transmission for metering systems; 7. Furrow opening system; 8. Rotary tillage shaft; 9. Cover for rotovator (adapted from Krupnik et al., 2013).

Bed planting has long been a subject of research in South Asia and Bangladesh. Bed planting machinery (Fig. 1A) can be attached to two-wheeled tractors and used to make long beds alternating with furrows (sometimes referred to as ridges or raised beds) onto which crops can be sown (Talukder et al., 2011; Krupnik et al., 2013). The bed planter consists of several major components including a bed former, seedbox and metering system, fertilizer boxes and metering system, power transmission, rotary tillage systems, and furrow openers and seed dropping mechanisms (Fig. 2A and B). The furrows facilitate cross-field surface irrigation and are more time and water use efficient than traditional flood irrigation (Devkota et al., 2013). Bed planting – especially when a permanent part of conservation agricultural practices – has been shown to reduce production costs, maintain or increase yields, while also improving soil structure and reducing greenhouse gas emissions and arsenic contamination of crops (Duxbury et al., 2007; Verachtert et al., 2009; Aravindakshan et al., 2015; Gathala et al., 2015, 2016). Devkota et al. (2015) also showed that specific bed architectures can be used to reduce the impact of soil salinity on yield. Where excessive rainfall is a concern, beds can also assist in alleviating waterlogging stress (Fig. 1B). Beds made by two-wheel tractor attachable implements are trapezoidal in shape, and are usually 50–60 cm, with a total length of 75–90 cm from furrow to furrow base. One row of maize or legumes, or two rows of wheat or rice can typically be established on one bed (Talukder et al., 2011).

Although bed planters had been researched for over a decade in Bangladesh, JE was one of the first workshops to pro-actively adopt two-wheel tractor attachable bed planters on an independent commercial basis. One of the challenges faced by JE was construction of the bed planter's seed box, seed meter, and seed delivery mechanism, which are detailed by Krupnik et al. (2013), and usually make use of inclined plates to meter seed. Precise construction and assembly of the bed planter's seed metering system, which is of relevance to assure adequate crop establishment to maintain yields and farmers' satisfaction, is used as the case study subject of the remainder of this paper.

## 3 Methods

### 3.1 Research process

We engaged with JE over a period of two months from June to July in 2015. The PAR process consisted of the following steps, which will be further detailed as follows. We (1) first visited the factory site to prepare a scale diagram of current equipment layout (section 3.2). Following this, we (2) collaborated with JE to create breakdowns of the seedbed planter components, sub-assemblies and assemblies (section 3.3). This enabled the cooperative development of (3) workflow diagrams for the fabrication and assembly of the seedbed planter assemblies (section 3.4), and also factory layout options including comparisons without constraints, with electrical location constraints, and with material location constraints (section 3.5). We also (4) evaluated the use frequency of each tool and the total distance traveled by the components and sub-assemblies using from-to charts and measured handling distance to rank order factory layout options (section 3.6). These steps were followed by (5) a convening of JE staff to consider functionality and safety issues (section 3.7), after which we (6) facilitated discussions to generate new insights and preferences for new proposed layouts (section 3.8).

### 3.2 Description of Janata Engineering

**Fig. 3:**
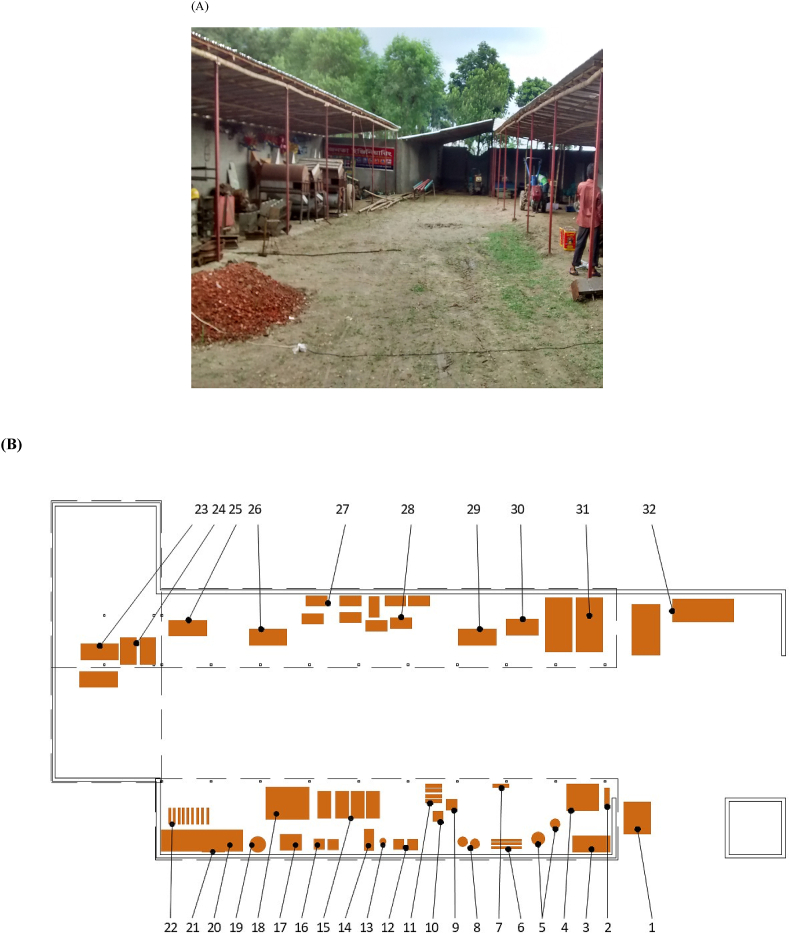
(A) Baseline Janata Engineering Workshop. (B) Schematic drawing: 1 Tuk Tuk; 2 Press; 3 Lathe; 4 Break Table; 5 Parts; 6 Stock; 7 Seed bin; 8 Drill press; 9 Arc welder; 10 Tillage Shaft; 11 Seed bins; 12 Arc welder; 13 Axle connection; 14 Mill and Lathe; 15 Rice hullers; 16 Spot welder; 17 Thresher; 18 Trailer; 19 Wire spool; 20 Work table base; 21 Corn kernel removers; 22 Rollers; 23 Tractors; 24 Attachments; 25 Tractor; 26 Tractor; 27 Tillers; 28 Tillers; 29 Tractor; 30 Table; 31 Trailers; 32 Bricks.

The production operations of JE, located in Chuadanga, in the southwest of Bangladesh, were established in the mid 2000s. In mid-2015, when this research was conducted, the workshop consisted of an earthen floor with sections covered by corrugated tin roofing (Fig. 3A). Total floor space was approximately 610 m^2^, laid out in an L-shape. The workshop had access to reliable electricity and was sub-divided into sections used for storage and construction of a variety of agricultural machineries (Fig. 3B). Like most emerging machinery manufacturers in Bangladesh, the workshop did however not have clear allocations for dedicated spaces for its various functions. These include business operations, fabrication, assembly and testing, inventory storage, space for workers to take breaks, and storage for the employees’ belongings during work hours.

### 3.3 Bed planter seed meter assembly

**Fig. 4:**
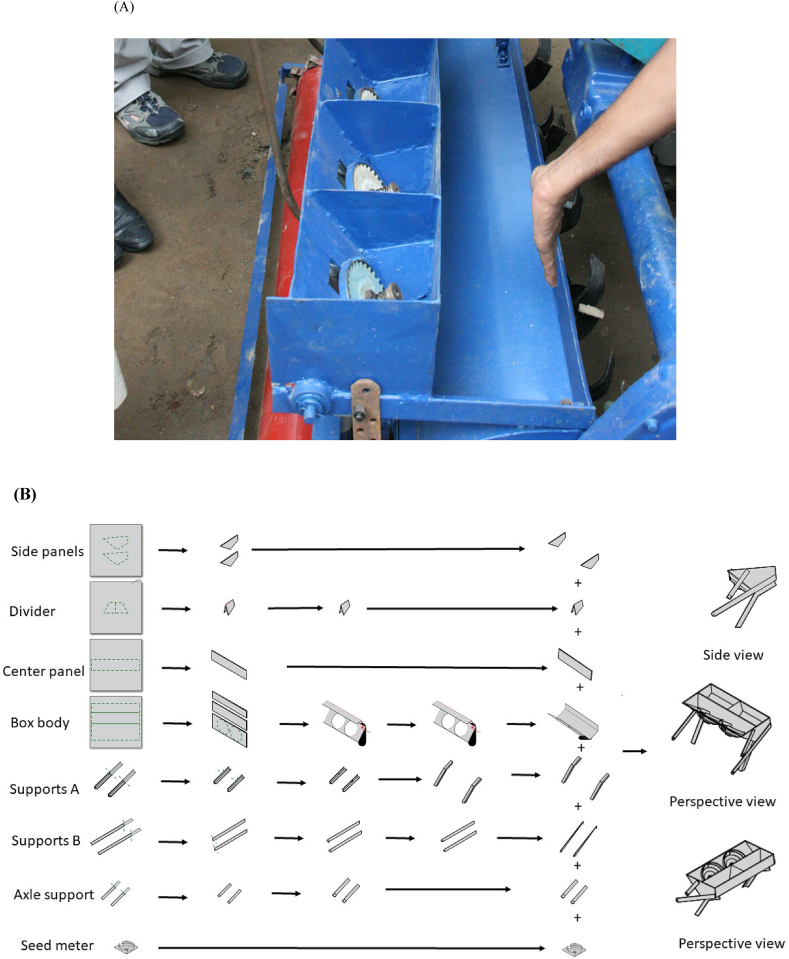
(A) Generic example of a seed box with inclined plate seed mechanism. (B) Assembly drawing: from left to right, the seed box assembly process begins with the raw materials and proceeds through the fabrication steps to the finished components, which are combined to form the subassembly.

This paper focusses on bed planter seed meter manufacturing (Fig. 4A), as a representative sub-assembly that will be used to illustrate the principles by which small-scale machinery manufacturing can be laid out to optimize the production process. Fig. 4B details the current fabrication process for the seed box as practiced by JE prior to the initiation of our PAR research process. Each component begins as a raw material selection, such as steel sheet or angle bar, which is physically shaped by tools, such as shears, lathe, and welder. The product is a component of a subassembly that is a part of the bed planter assembly. Prior to our research, most seed meters produced by JE lacked standardization and quality control processes; each meter was slightly different in size, shape, and architecture, resulting in variable and sub-optimal performance when used in farmers’ fields. Following requests by JE to assist in improvement of their seed meter manufacturing process, the research reported in this paper was undertaken to design and vet different options for improved factory layout and work flow process, with the ultimate goal of improving bed planter seed meter manufacturing.

### 3.4 Workflow diagrams

**Fig. 5:**
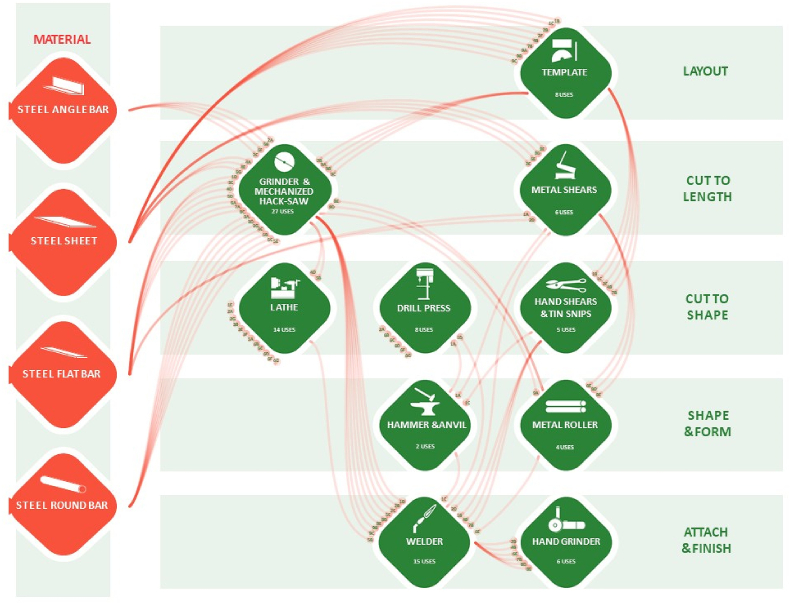
Tool Usage and Flow Diagram for Sub-assemblies: the materials are represented by the red diamonds; the flow of the materials is represented by the red lines; the tools are represented by the green diamonds; and the use of the tools is represented by the endpoints of the red lines.

**Fig. 6:**
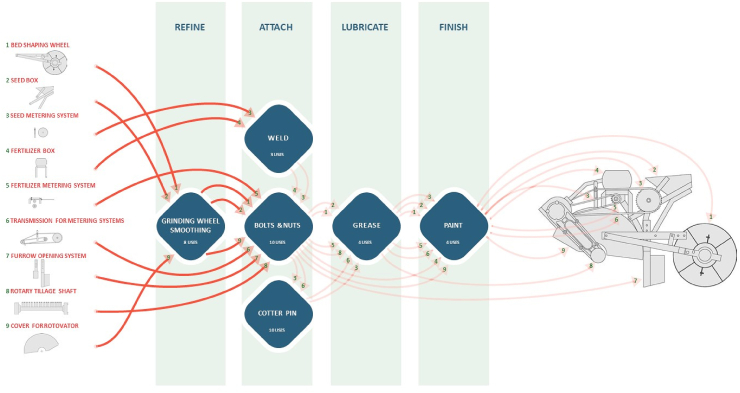
Tool Usage and Workflow for Final Assembly: Subassemblies are listed in the left column; a subassembly's path through the finishing process is represented by the red lines; the tool or product used in joining or finishing is represented by the blue diamonds; and the use of those tools or products is represented by the endpoints of the red lines.

Workflow diagrams were developed from the analysis of the current fabrication and assembly processes as a baseline on which to improve. These are shown in Fig. 5, Fig. 6, and will be discussed in Section 4.

### 3.5 Factory layout options

**Fig. 7:**
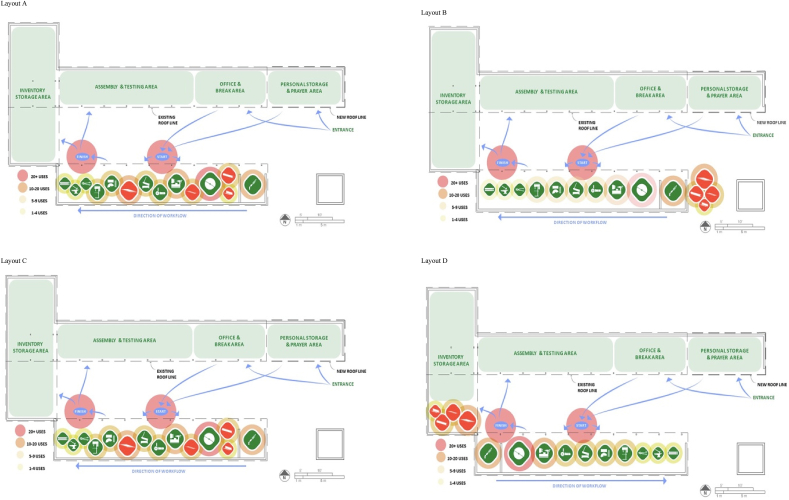
Proposed workshop layouts.

Using the workflow diagrams, different factory layouts were created to provide options accounting for efficient production based upon three different sets of constraints: (a) without electrical or material location constraints, (b) with electrical location constraints, and (c) with material location constraints. These are shown in Fig. 7, which will be discussed in Section 4.

### 3.6 Frequency of operations and from-to diagrams

Based on the collaboratively identified alternative workshop and work flow configurations, we analyzed the resulting use frequency of each tool needed in the fabrication process of the bed planter. The overall productivity of a workshop is improved by several factors including reducing labor, lowering chances of product damage, and increasing throughput speed. Lowering overall handling distance directly affects and reduces labor, probability of product damage, and throughput which, in turn, lowers costs. The best layout minimizes handling distance and cost. A travel or “from-to” chart accounts for these factors by tracking two sets of data: usage frequency of and distance between each material and tool in a potential layout (Riggs, 1987). The results are shown in Gregg (2015). These data were used to calculate and compare travel distances for the seed bed planter assembly. The ‘start’ and ‘finish’ points from a particular piece of manufacturing equipment are necessary constants for the purpose of gathering and analysis of data. These points serve as fixed locations from which each component fabrication begins and ends and are shown in Fig. 7. Based on recommendations from JE staff, we utilized the welding machine, which is the most frequently used piece of equipment in bed planter manufacturing, as the start point in our travel chart analyses.

These two sets of data were multiplied to provide the total distance traveled between each pair of materials or tools. The sum of all distances is the total distance traveled or total handling distance for that workshop layout. “From-to” charts were created for each proposed layout are shown in Table 1 and will be discussed in Section 4. We also assessed workspace efficiency, measured as the handling distance for bed planter sub-assemblies as calculated in our travel charts.

**Table 1:** Travel Charts tabulate the various distances traveled (meters) by material between points in the manufacturing facility. Numbers in boxes represent the total distances traveled by each component, which results by multiplying the frequency of each travel between operations by its distance. For example, in Layout A, Steel sheet (3) moves from IN to Template (6) for 16 total meters, then to Metal shear (7) for 7.32 total meters, and then to Grinder/Hack saw (8) for 26.6 total meters, summing to a motion of 49.9 total meters. Refer to Fig. 5, Fig. 6 for the order of movement.

Layout A																			
BED PLANTER	IN	1	2	3	4	5	6	7	8	9	10	11	12	13	14	15	16		TOTAL
IN		0.33	12.4	69.3	75.6	106													264
OUTSOURCED PART 1										23.2					71.5				94.6
STEEL ANGLE BAR 2									2.29										2.3
STEEL SHEET 3							16	7.32	26.6										49.9
STEEL FLAT BAR 4								18.8	14										32.8
STEEL ROUND BAR 5									14.6										14.6
TEMPLATE 6									59.7		11.3								71.0
METAL SHEAR 7												14.7	17.4	7.06			8.36		47.5
GRINDER/HACK-SAW 8										13.8		18.9		36.2			257		326
LATHE 9														4.78			168		173
HAND SHEARS 10													3.12	38			3.43		44.6
METAL ROLLER 11									37.9								2.54		40.4
HAMMER & ANVIL 12										13.4				15.7	57				86.1
WELDER 13															17	29.6	137		184
DRILL PRESS 14										87.8							6.25		94.0
HAND GRINDER 15																	31.5		31.5
FINISH 16																			0

TOTAL	0	0.33	12.4	69.3	75.6	106	16.0	26.1	155	138	11.3	33.6	20.5	102	145	29.6	614		1556

Layout B																			
IN		0.33	13.5	203	108	122													446
OUTSOURCED PART 1										23.2					71.5				94.6
STEEL ANGLE BAR 2									23.6										23.6
STEEL SHEET 3							80	48.5	47.1										176
STEEL FLAT BAR 4								24.2	141										166
STEEL ROUND BAR 5									212										212
TEMPLATE 6									59.7		11.3								71.0
METAL SHEAR 7												14.7	17.4	7.06			8.36		47.5
GRINDER/HACK-SAW 8										13.8		18.9		36.2			257		326
LATHE 9														4.78			168		173
HAND SHEARS 10													3.12	38			3.43		44.6
METAL ROLLER 11									37.9								2.54		40.4
HAMMER & ANVIL 12										13.4				15.7	1.45				30.6
WELDER 13															17	29.6	137		184
DRILL PRESS 14										87.8							6.25		94.0
HAND GRINDER 15																	31.5		31.5
FINISH 16																			0

TOTAL	0	0.33	13.5	203	108	122	80.0	72.7	522	138	11.3	33.6	20.5	101.8	89.9	29.6	614		2160

Layout C																			
BED PLANTER	IN	1	2	3	4	5	6	7	8	9	10	11	12	13	14	15	16		TOTAL
IN		0.33	13.9	209	111	125													460
OUTSOURCED PART 1										23.2					1.82				25.0
STEEL ANGLE BAR 2									3.94										3.9
STEEL SHEET 3							170	62.1	7.87										240
STEEL FLAT BAR 4								31	23.6										54.7
STEEL ROUND BAR 5									35.4										35.4
TEMPLATE 6									59.7		11.3								71.0
METAL SHEAR 7												14.7	17.4	7.06			8.36		47.5
GRINDER/HACK-SAW 8										13.8		18.9		36.2			257		326
LATHE 9														4.78			168		173
HAND SHEARS 10													3.12	38			3.43		44.6
METAL ROLLER 11									37.9								2.54		40.4
HAMMER & ANVIL 12										13.4				15.7	1.45				30.6
WELDER 13															17	29.6	137		184
DRILL PRESS 14										87.8							6.25		94.0
HAND GRINDER 15																	31.5		31.5
FINISH 16																			0

TOTAL	0	0.33	13.9	209	111	125	170	93.1	169	138	11.3	33.6	20.5	102	20.3	29.6	614		1860

Layout D																			
BED PLANTER	IN	1	2	3	4	5	6	7	8	9	10	11	12	13	14	15	16		TOTAL
IN		0.33	12.4	69.3	75.6	106													264
OUTSOURCED PART 1										23.2					71.5				94.6
STEEL ANGLE BAR 2									2.29										2.3
STEEL SHEET 3							16	7.32	26.6										49.9
STEEL FLAT BAR 4								18.8	14										32.8
STEEL ROUND BAR 5									14.6										14.6
TEMPLATE 6									59.7		11.3								71.0
METAL SHEAR 7												14.7	17.4	7.06			8.36		47.5
GRINDER/HACK-SAW 8										13.8		18.9		36.2			257		326
LATHE 9														4.78			168		173
HAND SHEARS 10													3.12	38			3.43		44.6
METAL ROLLER 11									37.9								2.54		40.4
HAMMER & ANVIL 12										13.4				15.7	1.45				30.6
WELDER 13															17	29.6	137		184
DRILL PRESS 14										87.8							6.25		94.0
HAND GRINDER 15																	57.5		57.5
FINISH 16																			0

TOTAL	0	0.33	12.4	69.3	75.6	106	16.0	26.1	155	138	11.3	33.6	20.5	102	89.9	29.6	640		1526

### 3.7 Design evaluation by JE staff focus group

We convened JE staff as a focus group to provide feedback on the functionality and safety included ground clearance to reduce hazards and injuries, location of where the material deliveries would be offloaded, and location where those materials would be most accessible during hours of operation and secure during hours when the facility is inactive. This feedback is provided in Table 2 and will be discussed in Section 4.

**Table 2:** Worker responses to questions on bed planter and seed meter manufacturing processes at Janata Engineering (JE).

Would JE benefit from implementation of Layout A? Why or why not?
• Yes, materials easier to get and handle. The problem would be maintaining shop discipline in ensuring materials were properly stored out of the way, or ensuring walking paths are maintained. This will be the most dangerous option, without proper safeguards or training in place.
Would JE benefit from implementation of Layout B? Why or why not?
• Found Layouts B and C to be quite similar and less useful for these metrics. • Yes, this is the safest option because it puts the loudest, dirtiest equipment closest to the outside. It also keeps the materials protected at the inside. This is the safest option, but will also be the most arduous for the workers because everything is hand carried between tools.
Would JE benefit from implementation of Layout C? Why or why not?
• Found layouts C and D to be quite similar and less useful for these metrics. • Yes, unloading and loading of materials is easiest with this option. However it puts the materials in an area too exposed to the outside, and is very close to the loud, high usage equipment. Additional walls or roofs would have to be constructed to isolate noise and the outside elements from workers retrieving materials.
Would JE benefit from implementation of Layout D? Why or why not?
• This one tended to be preferred as well because it is compact, and raw materials are kept near general storage. • No, same answer as for Layout C, but has increased difficulty of unloading and loading materials.

### 3.8 Delivery to JE

Finally, recommendations were fed-back to JE in the form of a redesign of the workshop workflow through layout of materials, tools, assembly, testing, and facilities areas.

## 4 Results and discussion

### 4.1 Workflow and equipment allocation

Well-designed allocation of space and equipment layout can increase overall productivity by reducing labor, lowering product damage, increasing throughput speed, easing future incorporation of new processes, and improving safety and morale (Adam, 1989). The foundation of a well-designed layout begins with space allocation. Managing the space is the first step in developing an efficient workflow in and between each designated area. Action research results indicated that the managers of the JE workshop prioritize six primary functions: (1) parts fabrication with tools and raw materials, (2) assembly and testing, (3) inventory storage, (4) business operations and (5) space for taking breaks, and (6) personal storage.

Diagrams of the tool usage and workflow graphically represent the various actions performed and the tools and materials used in the JE workshop to fabricate bed planter components and subassemblies (Fig. 5). Quantifying the use of each material and tool is part of the process to develop the data used to improve workshop design and layout. Similarly, a diagram of the tool usage and workflow for final assembly was drawn to represent the different actions performed and the tools and materials used in the JE workshop to assemble the sub-assembly components into a complete bed planter (Fig. 6). The use of a grinding wheel and welder are operations that overlap with the sub-assemblies fabrication workflow because the machines used in the subassemblies fabrication may also be used for the bed planter final assembly.

### 4.2 Alternative workshop layouts

The predefined footprint of the JE workshop dictates that the locations for the primary functions surround a central open space (Fig. 3B). This courtyard style layout is typical, providing a long perimeter that has protection from the sun but still provides ample natural light by which to work. Fig. 7 shows the proposed layouts of a redesigned workshop. With the areas of highest activity on the north and south of the workshop, they receive the most shade and light throughout the day. The new layouts depicted in Fig. 7A–D also allow for easy communication and movement between the areas. With the new, clear allocation of space, the workshop will be more user friendly. Upon entering the workshop, the new organization of space provides an area to the north for storage of personal belongings, as well as a clear area for workers to partake in their daily prayers. This design was proposed following indications that the JE employees remain in the workshop for meals and routine religious prayer that may otherwise require the time and energy to make a trip home. The adjacent area to the west is designed to be equipped with tables for business operations and breaks for rest and meals. Next, the area for assembly, testing, and repairs is located for easy monitoring from the business operations area and access to the inventory storage area, where parts, subassemblies, finished seeders, etc. are stored out of the way of areas of higher activity.

Travel chart results for each of the layouts shown in Table 1 for each alternative workshop layout (A-D) shown in Fig. 7. The fabrication area on the south side of the workshop has the highest level of activity and the greatest opportunity for improved efficiency. The proposed layout shown in Fig. 7D offers the best balance between efficiency, functionality, and safety of the four proposed designs.

### 4.3 Design evaluation by experts

Table 2 present the results of evaluations by JE staff. Layout A was perceived to be the most potentially efficient, functional, and profitable; however, this layout also received the lowest ranking in perceived safety. The issue with safety for Layout A arises from the integration of materials storage in the workflow path between machines and tools. As safety is an integral part of a strong work environment, Layout A may therefore not be the best design option to implement at JE.

Layouts B and C were considered as alternatives to, but not quite as strong as, layout D in terms of efficiency, functionality, and profitability. Layout B was also considered the safest option by a slight margin. Therefore, layout D appeared to be the most preferable design of the four options for implementation at JE. Feedback on layout D also included preference for the compact design and for the location of raw material storage adjacent to general storage.

## 5 Final design selection and discussion

Janata Engineering has been growing steadily since the initiation of this design exercise. Based on this and other technical back-stopping, they have expanded into the manufacture of additional agricultural and also non-agricultural products, such as bicycle wheel spokes. In response to these emerging business opportunities, they no longer focus exclusively on two-wheel tractor-based crop establishment equipment and as such have modified their factory accordingly. For these reasons, the specific designs discussed in this paper are no longer applicable. This is however not an inherent limitation of this research; rather, our efforts aimed at developing *processes* and *procedures* that are the core plank of PAR (Bawden, 1990; Miskovic and Hoop, 2006) that can assist in the rationalization of workplace design to improve productivity and safety. Although JE did not implement the entirety of the PAR-based design outputs discussed in this paper, during the iterative PAR phases they strongly indicated the benefits the research and discussion processes as being influential in how they thought about designing and implementing new factory layouts and product manufacturing processes.

For example, JE is currently building a third factory opposite the second factory to accommodate the production of additional products. Janata Engineering has also expressed interest in using PAR processes to assist in the design and layout of their third factory, while also making initial use of the insights derived from the work described in this paper for partial redesign of their factory floors. This appears to be due to their perception that the designs produced by PAR processes were more informed, customized, and ultimately better in terms of efficiency, safety and functionality when compared to the status quo that lacked input from factory workers, technical experts, and failed to make use of best manufacturing practices. Janata Engineering currently does not have access to or sufficient expertise to utilize computer-based design tools, such as CAD/CAM, although capacity development in this area for JE and similar manufacturers – which are common in Bangladesh –would enable the use of more sophisticated design systems. That is not to say that these approaches are a suitable substitute for PAR; rather, they could be complementary where computer-based design processes incorporate sufficient feedback and iterative redesign and improvement with the involvement of employees, managers, and factory owners.

The Bangladesh SME agricultural machinery manufacturing sector is an example of a vibrant, dynamic community that is working hard to improve its human and institutional capacity, yet currently lacks the technical skills (e.g., numeracy, metrology, engineering, computer aided design and manufacturing, and supporting testing laboratories) and manufacturing machinery required to move to the next level of quality and efficiency. As initially demonstrated by this proof-of-concept paper, PAR can support the improvement up to and throughout the period when more advanced technical methods are required to design future manufacturing processes.

The process outlined in this paper provides a framework for the design of workshops that can be adapted and utilized in other developing countries. Factory design schemes for developing countries tend to be focused on conventional goals of technical efficiency, rather than incorporating employee's ideas and contributions in terms of functionality, safety, and human interactions. They also tend to be directed toward large companies with resources and technical skills (Utkigar, 2009). As a result, they may not always be appropriate for small enterprises that have limited technical knowledge and capital resources. As the literature is generally silent on this topic, the current study is, to our knowledge, the first reported to use PAR as applied to design methods and industrial manufacturing. The method is relatively easy to follow and implement with basic tools and mathematical literacy, although familiarity with PAR and strong social and facilitation skills are needed alongside technical competencies. As such, the methods could have considerable usefulness and can be applied in a number of settings within developing countries – not only limited to agricultural machinery – as they increase their light engineering and spare parts capacity development. As discussed above, this approach does not exclude computer-based factory layout and simulation tools; rather, PAR can be used as a complement for these processes.

## 6 Conclusions and implications

This paper presented a process using participatory action research as applied to the design and optimization of agricultural machinery workshops for developing countries, using Janata Engineering and the manufacturing and assembly of two-wheel tractor attachable bed planters in Bangladesh as a case study example. The steps include iterative interactions with the manufacturer to understand the component processes used in manufacturing and assembling the bed planter product, followed by the determination of improved fabrication and assembly operations required to make the components and assemble them, after which alternative workshop layout options were developed with the participation of JE staff. These designs were based upon considerations of importance to workshop workers, including locations of business operations, fabrication, assembly and testing, inventory storage, space for taking breaks, and storage for the employees’ belongings during work hours. Four alternative layout designs for the fabrication and assembly area were generated, which were subsequently analyzed using travel charts and through focus group evaluation with JE staff. This process is useful for the design and optimization of small-scale agricultural machinery workshops in Bangladesh and can be applied to improve agricultural machinery manufacturing in similar developing country contexts.

## Declaration of competing interest

This research was supported by the Cereal Systems Initiative for South Asia (CSISA) Phase II and the CSISA-Mechanization and Irrigation projects funded by the 10.13039/100000865Bill and Melinda Gates Foundation (10.13039/100000865BMGF) and 10.13039/100000200USAID. Additional support was provided through the MAIZE CGIAR Research Program. The results of this research do not necessarily reflect the views of USAID, the United States Government, or the BMGF.
